# Identification of lipocalin-2 as a PKCδ phosphorylation substrate in neutrophils

**DOI:** 10.1186/s12929-015-0129-z

**Published:** 2015-03-20

**Authors:** Yi-Chinn Weng, Guona Wang, Robert O Messing, Wen-Hai Chou

**Affiliations:** Department of Biological Sciences, School of Biomedical Sciences, Kent State University, Kent, OH 44224 USA; Department of Neurology, University of California, San Francisco, CA 94608 USA; Division of Pharmacology and Toxicology, College of Pharmacy, The University of Texas at Austin, Austin, TX 78712 USA

**Keywords:** PKC, Stroke, Neutrophil, Lipocalin-2, Phosphorylation

## Abstract

**Background:**

PKCδ expressed in neutrophils is implicated in promoting reperfusion injury after ischemic stroke. To understand the molecular and cellular actions of PKCδ, we employed a chemical-genetics approach to identify PKCδ substrates in neutrophils.

**Results:**

We recently generated knock-in mice endogenously expressing analog-specific PKCδ (AS-PKCδ) that can utilize ATP analogs as phosphate donors. Using neutrophils isolated from the knock-in mice, we identified several PKCδ substrates, one of which was lipocalin-2 (LCN2), which is an iron-binding protein that can trigger apoptosis by reducing intracellular iron concentrations. We found that PKCδ phosphorylated LCN2 at T115 and this phosphorylation was reduced in *Prkcd*^*−/−*^ mice. PKCδ colocalized with LCN2 in resting and stimulated neutrophils. LCN2 release from neutrophils after cerebral ischemia was reduced in PKCδ null mice.

**Conclusions:**

These findings suggest that PKCδ phosphorylates LCN2 and mediates its release from neutrophils during ischemia-reperfusion injury.

## Background

Ischemic stroke is a leading cause of mortality and disability in the United States [1]. Early reperfusion with thrombolytic agents can be effective in treating acute ischemic stroke, but may initiate and enhance inflammatory responses causing reperfusion injury [2]. After reperfusion, neutrophils adhere to cerebral vasculature, infiltrate ischemic brain tissues, and release free radicals and proteins that may exacerbate brain injury [3]. Our prior work indicates that PKCδ is necessary for neutrophil activation following ischemia and reperfusion. We found that *Prkcd*^*−/−*^ mice show a striking 70% reduction in brain injury after ischemic stroke [4]. This outcome was associated with reduced infiltration of neutrophils into infarcted tissue, as well as impaired neutrophil adhesion, migration, respiratory burst, and degranulation. To further understand the molecular and cellular mechanisms by which PKCδ contributes to reperfusion injury, we aimed to identify PKCδ substrates in neutrophils.

PKC constitutes a family of 10 serine-threonine kinases sharing a highly conserved catalytic domain [5]. Similarities in the catalytic domains of PKC isozymes make it difficult to identify the unique targets of an individual isozyme in the presence of related cellular kinases. A chemical-genetic approach has been developed to identify kinase substrates and specific kinase inhibitors by modifying the gatekeeper residue of catalytic domain to accept analogs of ATP and PP1 inhibitors that have low affinity for native kinases [6,7]. Using this approach we generated and characterized an analog-specific PKCδ (AS-PKCδ) [8-10]. AS-PKCδ shows kinase activity similar to wild type PKCδ, but can utilize N^6^-(benzyl)-ATP as a phosphate donor and is uniquely sensitive to inhibition by 1NA-PP1 [8-10]. To identify PKCδ substrates in neutrophils, we wanted to express AS-PKCδ in neutrophils, but because they are terminally differentiated and difficult to transfect, we instead used AS-PKCδ knock-in mice, which endogenously express AS-PKCδ [10]. Using neutrophils isolated from these knock-in mice, we identified lipocalin-2 (LCN2) as a PKCδ substrate. LCN2, also known as oncogene 24p3, siderocalin or neutrophil gelatinase-associated lipocalin (NGAL), was initially purified as a 25 kDa protein secreted from neutrophils [11]. Several pathological conditions including bacterial infection [12], renal ischemia [13], spinal cord injury [14], and ischemic stroke [15-17] trigger the release of LCN2. Here we found reduced release of LCN2 in *Prkcd*^*−/−*^ mice following cerebral ischemia, indicating an important role for PKCδ in LCN2 secretion.

## Methods

### Kinase assay

Neutrophils were isolated from bone marrow by Percoll density gradient centrifugation [4,18]. Neutrophils were lysed by freeze/thaw treatment in modified RIPA buffer containing 50 mM Tris–HCl pH 7.4, 150 mM NaCl, 1% NP-40, 5 mM EDTA, 5 mM EGTA, phosphatase inhibitor cocktails I and II (Sigma-Aldrich), and cOmplete^TM^ protease inhibitor cocktail (Roche), and were centrifuged at 20,000 g for 15 min at 4°C. The supernatant (100 μg proteins) was incubated in 60 μl of PKC reaction buffer containing 20 mM HEPES pH 7.4, 0.1 mM EGTA, 0.03% Triton X-100, and 10 mM MgCl_2_ at 27°C for 30 min with 1 mM GTP, 200 μM N^6^-(benzyl)-ATP-γS (Biolog), and 1 μM phorbol 12-myristate 13-acetate (PMA) (Sigma-Aldrich) to initiate the reaction. Control reactions without PMA, and reactions with 1 μM 1NA-PP1 (Calbiochem) to inhibit AS-PKCδ activity were included. The kinase reactions were stopped by adding 20 mM EDTA. Thiophosphorylated proteins were alkylated by incubation with 2.5 mM para-nitrobenzylmesylate (PNBM) (Epitomics) for 2 h at room temperature.

### Fractionation by MicroSol-IEF

Alkylated proteins were resolved by isoelectric focusing using a Zoom-IEF Fractionator (Invitrogen), according to the manufacturer’s protocol [19]. Alkylated samples were dissolved in IEF fractionation buffer containing 7 M urea, 2 M thiourea, 4% CHAPS (Invitrogen), 65 mM DTT, 1 mM EDTA, protease inhibitors cocktail (Roche), and 1 mM PMSF (Sigma-Aldrich), and centrifuged at 20,000 g for 60 min at 4°C. The supernatant was collected to determine the protein concentration by the Bradford protein assay using BSA as a standard and were adjusted with IEF fractionation buffer to 0.6 mg/mL. Aliquots of 650 μl were loaded in five different pH chambers (pH 3.0-4.6, pH 4.6-5.4, pH 5.4-6.2, pH 6.2-7.0, and pH 7.0-10.0) in the Zoom-IEF Fractionator, and processed sequentially at 100 V for 20 min, 200 V for 80 min and 600 V for 80 min. The fractionated samples from each chamber were analyzed by western blot analysis using rabbit monoclonal antibodies against the thiophosphate esters (1:10,000 dilution; Abcam) and HRP conjugated secondary antibodies (1:1,000 dilution; Jackson ImmunoResearch). Gel loading was assessed by western blot analysis with a mouse monoclonal anti-actin antibody (1:2,000 dilution; Sigma-Aldrich).

### MALDI-TOF mass spectrometry for protein identification

Protein bands of interest detected on western blots were excised from parallel Coomassie Blue stained gels and placed into microcentrifuge tubes. The gel slices were dried in a vacuum concentrator, rehydrated and digested in trypsin solution (12.5 ng/μl sequencing grade trypsin in freshly diluted 25 mM ammonium bicarbonate) overnight at 32°C. The liquid containing trypsinized peptides was extracted twice with 50 μl of 50% acetonitrile/2% TFA. The combined extracts were dried and resuspended in matrix solution (10 mg/ml 4-hydroxy-α-cyanocinnamic acid in 50% acetonitrile/0.1% TFA). Matrix-Assisted Laser Desorption/Ionization Time-of-Flight (MALDI-TOF) mass spectrometric analysis was performed on the digest using a PerSeptive Voyager DE-RP mass spectrometer in the linear mode by Applied Biomics, Hayward, CA. Detected peptides were analyzed by peptide mass fingerprinting using the Mascot search engine [20].

### Purification of recombinant LCN2

A plasmid (kindly provided by M. Green, U. Mass. Med. School) containing the cDNA sequence encoding the mouse Lcn2 gene in pGEX-2T (GE Healthcare) was expressed in *E. coli* BL21(DE3)pLysS cells (Invitrogen) [21]. GST-LCN2 fusion protein was purified by affinity chromatography using Glutathione-Sepharose 4B beads (GE Healthcare). Purified GST-LCN2 was incubated with thrombin-agarose (Sigma-Aldrich) to remove the GST tag. The supernatant containing cleaved GST and LCN2 was incubated with Glutathione-Sepharose to absorb the GST. The supernatant containing only LCN2 was collected for *in vitro* kinase assays.

### *In vitro* kinase assay

*In vitro* phosphorylation of purified LCN2 by recombinant PKCδ (Invitrogen) was performed as described [22]. The kinase reaction was initiated at 37°C by adding recombinant LCN2 and [γ-^32^P]ATP. At different time points, the reaction mixture was stopped by the addition of SDS sample buffer. Proteins were separated on NuPAGE Bis-Tris gels (Invitrogen) and stained using SimplyBlue (Invitrogen). Phosphorylated LCN2 was detected by phosphorimaging (Typhoon 9410, Amersham Bioscience).

### Mapping phosphorylation sites by tandem mass spectrometry

To identify the PKCδ phosphorylation sites, LCN2 protein was phosphorylated *in vitro* by PKCδ using 0.5 mM non-radiolabeled ATP for 4 h as described above. The reaction mixtures were fractionated by SDS-PAGE and stained with SimplyBlue (Invitrogen). Gel slices containing the phosphorylated LCN2 were treated with trypsin, and the resulting peptide mixtures were analyzed by nano-liquid chromatography-mass spectrometry/mass spectrometry (nano-LC-MS/MS) serviced at the Protein Chemistry Center, UT Southwestern Medical Center. Samples from the digests were analyzed by nano-LC-MS/MS using a LC-Packings HPLC (Dionex) coupled to a QStar XL mass spectrometer (Applied Biosystems). Data were searched against a home-built database that includes the LCN2 sequence. Four modifications were included in the database search: carbamidomethyl (C), oxidation (M), phospho (ST), and phospho (Y).

### Generation of LCN2 phosphorylation site mutants

LCN2 T115A was generated by site-directed mutagenesis using a QuikChange mutagenesis kit (Stratagene). The mouse Lcn2 cDNA subcloned into pGEX-2TK was used as a template to replace Thr-115 with Ala using the following primers: 5′-GCTCCAGGGCTGGCCAGTTCGCCCTGGGAAATATGCACAGG-3′ (forward) and 5′-CCTGTGCATATTTCCCAGGGCGAACTGGCCAGCCCTGGAGC-3′ (reverse). The coding region was sequenced to confirm error-free PCR and the T115A mutation. The LCN2 T115A protein was generated in *E. coli* BL21(DE3)pLysS cells and purified as described above.

### Detection of LCN2 T115 phosphorylation by phospho-specific antibody

A polyclonal rabbit anti-phospho-LCN2 (T115) antibody against a phosphopeptide ^110^RAGQF[pT]LGNMHR^121^ was generated and affinity-purified as a service at ProSci Inc. We optimized specificity for the targeted phospho-epitope using both negative and positive affinity purification methods, as described [23]. The phospho-LCN2 (T115) antibody (1:500 dilution) was used in western blotting to detect the phosphorylation of LCN2 in neutrophil lysates or LCN2 phosphorylated *in vitro* by PKCδ in the presence of non-radioactive ATP.

### Immunofluorescence

Neutrophils were plated on glass coverslips coated with 20% fetal calf serum for 10 min at 37°C. The coverslips with attached neutrophils were treated with or without 1 μM formyl-Met-Leu-Phe (fMLP) for 10 min at 37°C and fixed in 2% paraformaldehyde in PBS for 10 min at room temperature [24]. After permeabilization in 0.1% Triton X-100 in PBS for 5 min, neutrophils were blocked in 10% normal donkey serum (NDS), 0.2% BSA in PBS for 1 h and incubated with mouse anti-PKCδ antibody (1:200 dilution; BD) and goat anti-LCN2 antibody (1:200 dilution; R&D Systems) diluted in PBS containing 2% NDS and 0.2% BSA overnight at 4°C. After three washes with PBS, neutrophils were incubated with the appropriate Donkey fluorochrome-conjugated secondary antibodies (1:200 dilution; Jackson ImmunoResearch) and cover-slipped in mounting media containing DAPI (Vector Labs) to localize nuclei. Images were captured using Zeiss LSM 510 laser confocal microscope.

### Secretion of LCN2 in neutrophils

Neutrophils were stimulated with 1 μM of fMLP (Sigma-Aldrich) for 10, 20, 30, 60, and 120 min. Release and cell-associated LCN2 were analyzed by western blot analysis using anti-LCN2 antibody (R&D Systems).

### Cerebral ischemia and reperfusion

Global cerebral ischemia was induced by bilateral common carotid artery occlusion (BCCAO) without hypotension [25,26]. Mice weighing 25–35 g were anesthetized with 1.5% isoflurane in 30% O_2_/70% N_2_ using the V-10 Anesthesia system (VetEquip). Rectal temperature was maintained at 37 ± 0.5°C throughout the procedure by the TR-200 homeothermic temperature system (Fine Science Tools). Both common carotid arteries were carefully dissected away from the vagus nerves and occluded with Micro Serrefines (Fine Science Tools). Following 10 min of occlusion, the Micro Serrefines were removed from the CCA to induce reperfusion. All procedures were conducted in accordance with Institutional Animal Care and Use Committee policies.

### Collection of mouse serum

At different time points after BCCAO, mice were anesthetized with 5% isoflurane and euthanized by cervical dislocation. The blood was collected from the decapitated trunk and placed at room temperature for one hour. The blood was centrifuged at 2000 g for 20 min at room temperature, and the supernatant was collected as blood serum for western blot analysis [27].

### Statistical analysis

Quantitative data were expressed as mean ± SEM and analyzed using Prism 5.0 (GraphPad). Two-tailed, unpaired *t*-test or ANOVA with post hoc tests was used to determine statistical significance between means. The value of *p* less than 0.05 was considered to be statistically significant.

## Results

### Identification of PKCδ substrates in neutrophils

We found that only AS-PKCδ, but not wild type PKCδ, can use N^6^-(benzyl)-ATP in a kinase reaction with histone III as a substrate [8]. Previous studies have demonstrated the use of analog-specific kinases to identify substrates with ^32^P-labeled ATP analogs [28-33]. However, it remains challenging to isolate ^32^P-labeled substrates through conventional chromatography for identification by mass spectrometry. To facilitate the identification and purification of PKCδ substrates, we adopted an affinity tagging strategy to label substrates that can be recognized by a specific antibody [8]. First, an analog-specific kinase is used to thiophosphorylate substrates with N^6^-(benzyl)-ATP-γS. The thiophosphate groups are then alkylated by para-nitrobenzylmesylate (PNBM) to create thiophosphate ester epitopes that can be detected with the antibody.

**Figure 1:**
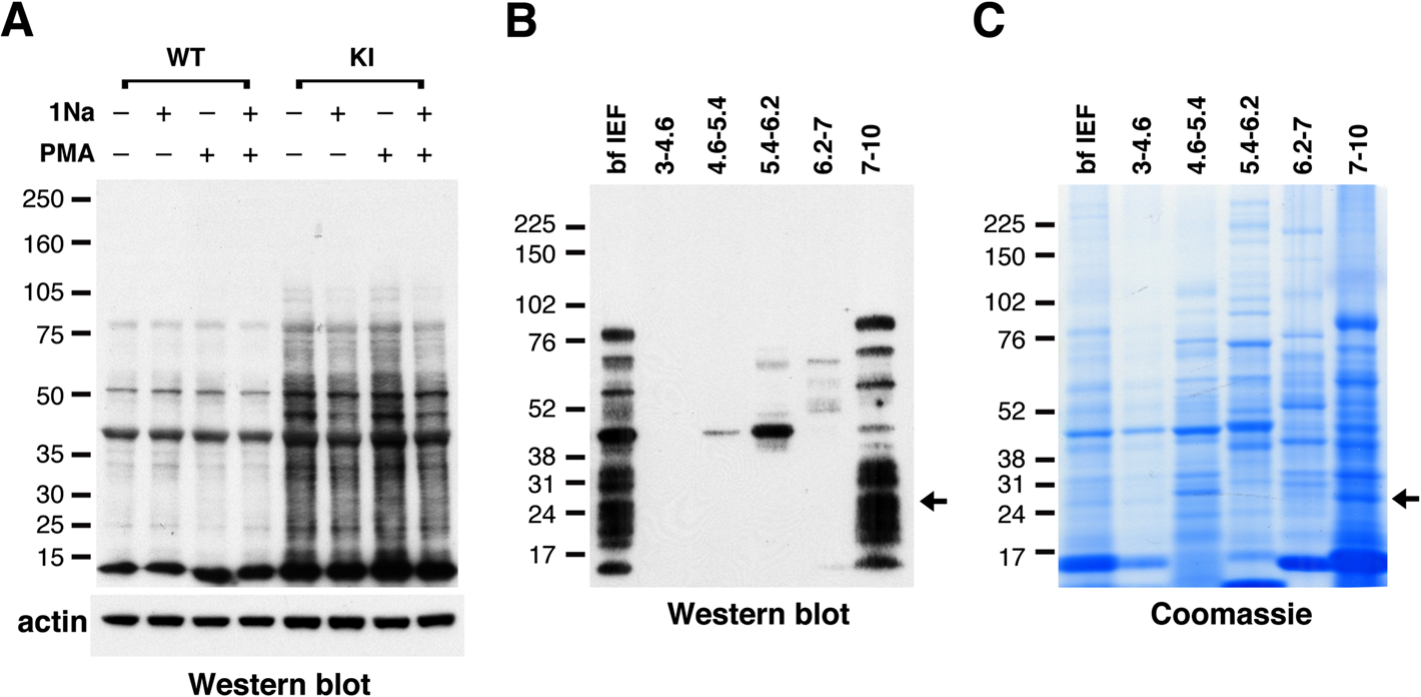
**Identification and purification of PKCδ substrates in neutrophils. (A)** Western blot with anti-thiophosphate ester antibody showing putative PKCδ substrates in neutrophil lysates from AS-PKCδ knock-in (KI) mice. The bottom panel shows β-actin immunoreactivity in the same samples as a loading control. **(B, C)** Identification of PKCδ substrates by MicroSol-IEF purification and mass spectrometry. AS-PKCδ neutrophil lysates were incubated with PMA and N^6^-(benzyl)-ATP-γS, and then thiophosphorylated proteins were alkylated with PNBM and separated by isoelectric focusing in 5 pools as shown. The proteins fractionated before (bf IEF) and after IEF were separated on parallel gels, one of which was subjected to western blot analysis using anti-thiophosphate ester antibody **(B)** and the other stained with Coomassie Blue **(C)**. The Coomassie-stained protein bands that matched with the immunoreactive bands were excised for analysis by mass spectrometry. The arrows indicate a protein band in the 7–10 pI pool that was identified as LCN2.

Using this method, we found several proteins were labeled in AS-PKCδ neutrophil lysates, but not in wild type lysates (Figure 1A). We purified some of these putative substrates by microscale solution isoelectric focusing (MicroSol-IEF) [19] and SDS-PAGE (Figure 1B and C). Coomassie blue–stained bands (Figure 1C) that matched immunoreactive bands (Figure 1B) were excised, and proteins in excised gels were identified by MALDI-TOF peptide mass fingerprint analysis. After a careful literature search to study known functions of these potential substrates and evaluate their potential contribution to understanding the role of PKCδ in stroke-induced reperfusion injury, we decided to focus on LCN2, a 25 kDa protein band identified in the fraction of pI 7–10 (Figure 1C). Previous studies have shown that LCN2 plays a vital role in apoptosis [21,34] and the plasma concentration of LCN2 is elevated in patients suffering from ischemic stroke [15-17]. Understanding the role of PKCδ-LCN2 signaling events may reveal an unidentified neural-immune interaction contributing to the induction of neuronal apoptosis.

### Identification of LCN2 T115 as the PKCδ phosphorylation site

**Figure 2:**
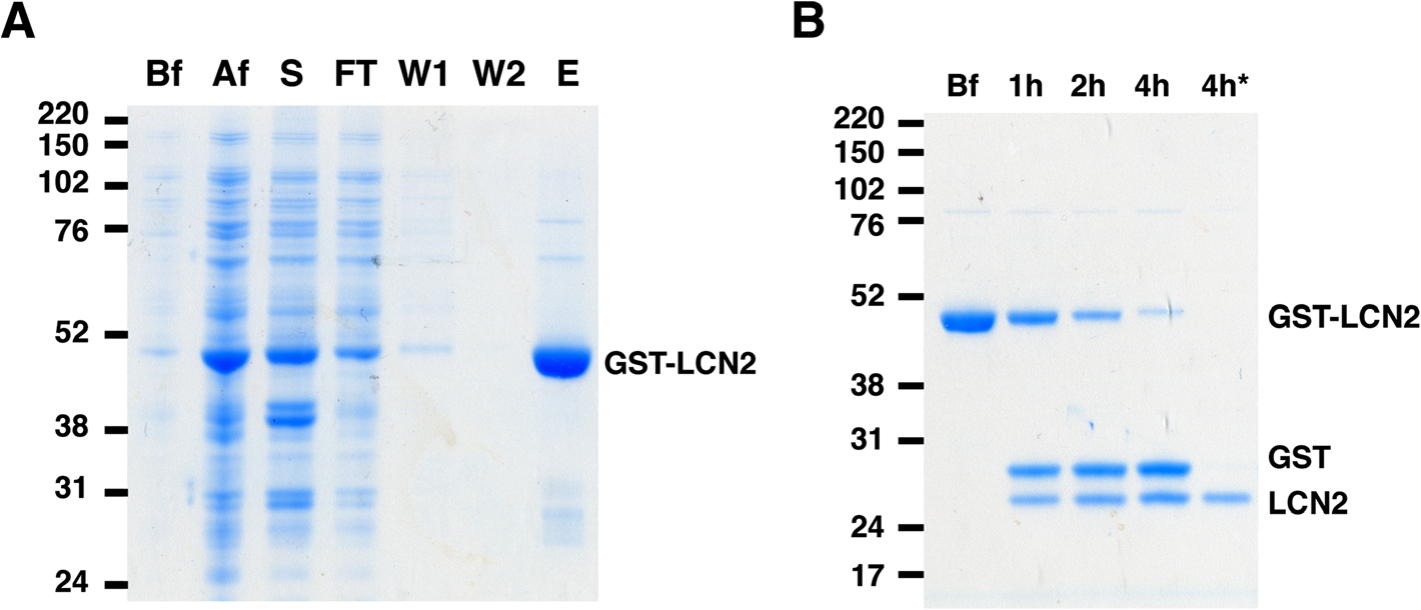
**Purification of LCN2. (A)** Recombinant GST-LCN2 was purified from *E. coli* BL21 by affinity chromatography using glutathione-sepharose 4B. Lysates from different purification steps were separated by SDS-PAGE: before (Bf) and after (Af) IPTG induction, supernatant after centrifugation (S), flow-through from column after loading supernatant (FT), 1st column wash (W1), 2nd column wash (W2), and elution of GST-LCN2 (E). **(B)** GST-LCN2 was digested by thrombin to remove the GST tag. GST-LCN2 was incubated with Thrombin-agarose (Sigma) at RT for up to 4 h. After 4 h incubation, the cleaved GST was absorbed by glutathione-sepharose. The supernatant containing only LCN2 (4 h*) was collected.

**Figure 3:**
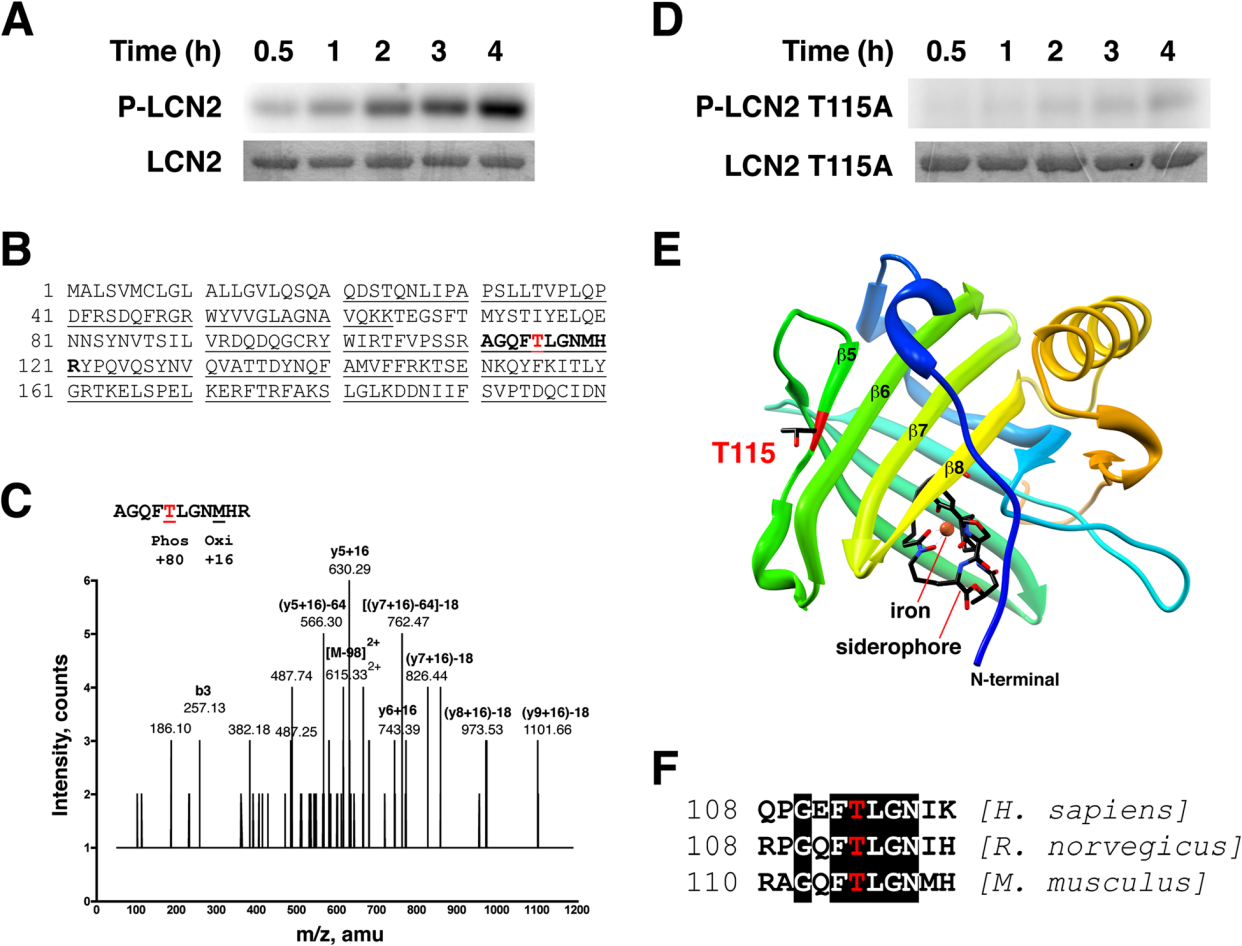
**PKCδ phosphorylates LCN2 at T115** ***in vitro*** **. (A)** Recombinant LCN2 proteins were subjected to *in vitro* kinase assays by PKCδ for up to 4 h. Representative autoradiographs of phosphorylated LCN2 (P-LCN2) are shown in the upper panel. Scanned images of Coomassie blue-stained gels (LCN2) are in the lower panel. **(B)** Analysis of LCN2 phosphorylation by PKCδ using mass spectrometry. LCN2 was phosphorylated by PKCδ and digested with trypsin. Fragments identified by nano-LC-MS/MS are underlined. The phosphorylated form of peptide AGQF[T]LGNMHR (in bold, amino acids 111–121) was detected. **(C)** Mass spectrometry spectrum. Identification of the phosphopeptide AGQF[T]LGNMHR (Mr = 1326.57, m/z = 615.33) by tandem MS indicating that the peptide was phosphorylated at T115. **(D)** Recombinant LCN2 T115A proteins were subjected to *in vitro* kinase assays by PKCδ for up to 4 h. **(E)** Crystal structure of LCN2 containing a siderophore (colored by atom type, N = blue, C = black, O = red) and an iron (orange). T115 with side chain shown is located in the β5 strand. **(F)** Sequence surrounding T115 (highlighted in red) from human, rat, and mouse LCN2 homologs. Conserved amino acids are surrounded by black boxes.

To investigate whether LCN2 is a direct substrate of PKCδ, we prepared recombinant LCN2 (Figure 2) and found that PKCδ phosphorylated LCN2 efficiently in *in vitro* kinase assays (Figure 3A). Nano-LC-MS/MS analysis of the phosphorylated and trypsin digested LCN2 identified a single phosphorylated peptide containing a threonine residue at 115 (in italics) (AGQF*T*LGNMHR; amino acids 111–121) (Figure 3B and C). To verify the PKCδ phosphorylation site, we generated a phosphorylation site mutant by mutating Thr-115 into Ala (T115A) and found that the T115A mutant is not phosphorylated by PKCδ (Figure 3D). A ribbon model of LCN2 (PDB number: 1X89) was generated using UCSF Chimera (http://www.cgl.ucsf.edu/chimera), and predicted that T115 is located in the solvent-accessible β5 strand (Figure 3E) [35]. T115 and surrounding residues are well conserved between human, rat, and mouse homologs of LCN2 (Figure 3 F) [36], suggesting a conserved role in function.

### Phosphorylation of LCN2 at T115 is reduced in neutrophils from *Prkcd*^*−/−*^ mice

**Figure 4:**
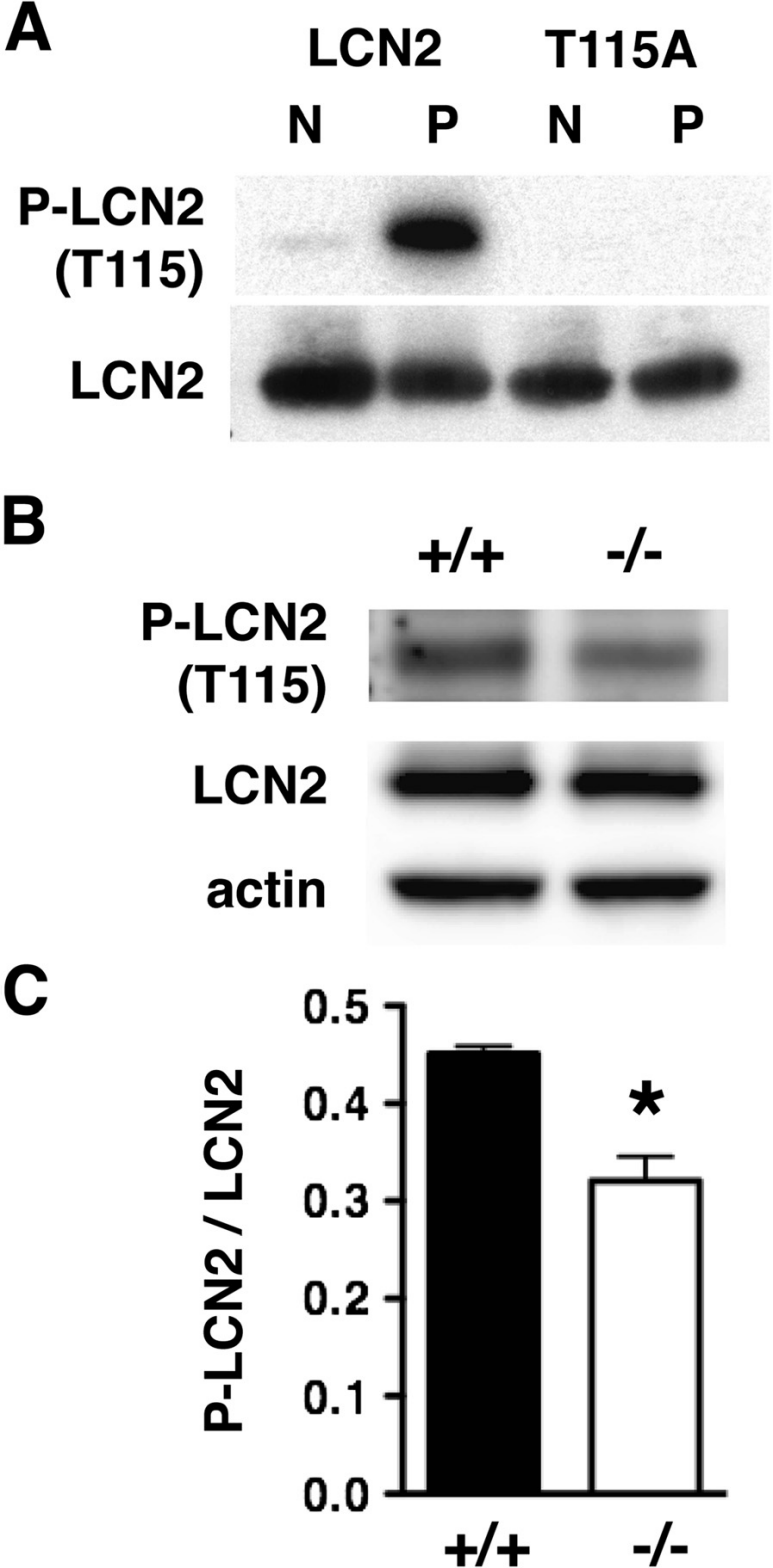
**PKCδ phosphorylation of LCN2 at T115 is reduced in neutrophils from** ***Prkcd*** ^***−/−***^ **mice. (A)** An affinity-purified rabbit anti-phospho-LCN2 (T115) antibody detected PKCδ phosphorylation of recombinant LCN2 (P), but not unphosphorylated LCN2 (N). The antibody was not immunoreactive against the LCN2 T115A mutant before or after incubation with PKCδ. (**B)** Representative western blot showing less immunoreactivity with the phospho-LCN2 (T115) antibody in neutrophil lysates from *Prkcd* ^*−/−*^ mice than in lysates from WT littermates. **(C)** The ratio of P-LCN2 (T115) to LCN2 immunoreactivity was significantly reduced in neutrophils from *Prkcd* ^*−/−*^ compared with *Prkcd* ^*+/+*^ mice (*n* = 3) (*p < 0.05, two-tailed *t* test). Error bars indicate SEM.

After identification of the PKCδ phosphorylation site *in vitro* (Figure 3), we investigated whether PKCδ phosphorylates LCN2 *in vivo*. We prepared an affinity-purified, phospho-specific antibody raised against a peptide containing phospho-T115. This anti-phospho-LCN2 (T115) antibody specifically detected recombinant LCN2 phosphorylated by PKCδ *in vitro*, but not unphosphorylated LCN2 or LCN2 T115A (Figure 4A). Anti-phospho-LCN2 (T115) immunoreactivity was reduced by 25% in *Prkcd*^*−/−*^ neutrophils compared with wild type neutrophils (Figure 4B and C), consistent with the prediction that PKCδ phosphorylates neutrophil LCN2 at T115 *in vivo*.

### PKCδ colocalizes with LCN2 in neutrophils

**Figure 5:**
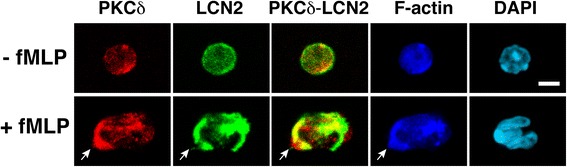
**Colocalization of PKCδ and LCN2 in neutrophils.** Neutrophils treated with or without fMLP were stained with specific antibodies against PKCδ (red) and LCN2 (green). Merged images (yellow) indicate colocalization of PKCδ and LCN2. Phalloidin staining (dark blue) revealed the reorganization of F-actin and polarization of neutrophils. An arrow indicates the front edge of the neutrophil that is enriched with F-actin. DAPI (light blue) was used to detect nuclei. Scale bars, 10 μm.

Since the integrity of subcellular compartments is lost during the preparation of cell lysates, it is important to demonstrate the colocalization of kinase and substrate in intact cells [37]. We performed immunofluorescence staining of neutrophils using antibodies against PKCδ and LCN2 (Figure 5). At baseline, PKCδ and LCN2 were diffusely distributed in the cell. Upon exposure to the fMLP chemoattractant, neutrophils took on a polarized morphology and initiated migration with F-actin enriched at the leading edge [38,39]. PKCδ was found mostly in the leading edge of polarized neutrophils [40] and LCN2 was detected at both poles. PKCδ and LCN2 colocalized with F-actin at the leading edge. The subcellular localization of LCN2 was previously determined by electron microscopic immunocytochemistry at high resolution, showing that LCN2 is present in the secondary vesicles (granules) and cytosol of neutrophils [41]. The presence of LCN2 in two different compartments is probably because LCN2 is a relatively small molecule (25 kDa) and can leak from vesicles (granules) into the cytosol [41]. PKCδ has been detected in the cytosol of neutrophils [42] as well as in granule fractions after stimulation with opsonized zymosan [43]. Based on previous studies and our current findings, we think that the colocalization of PKCδ and LCN2 supports their relationship as kinase and substrate in neutrophils.

### Release of LCN2 from neutrophils and after cerebral ischemia is reduced in *Prkcd*^*−/−*^ mice

**Figure 6:**
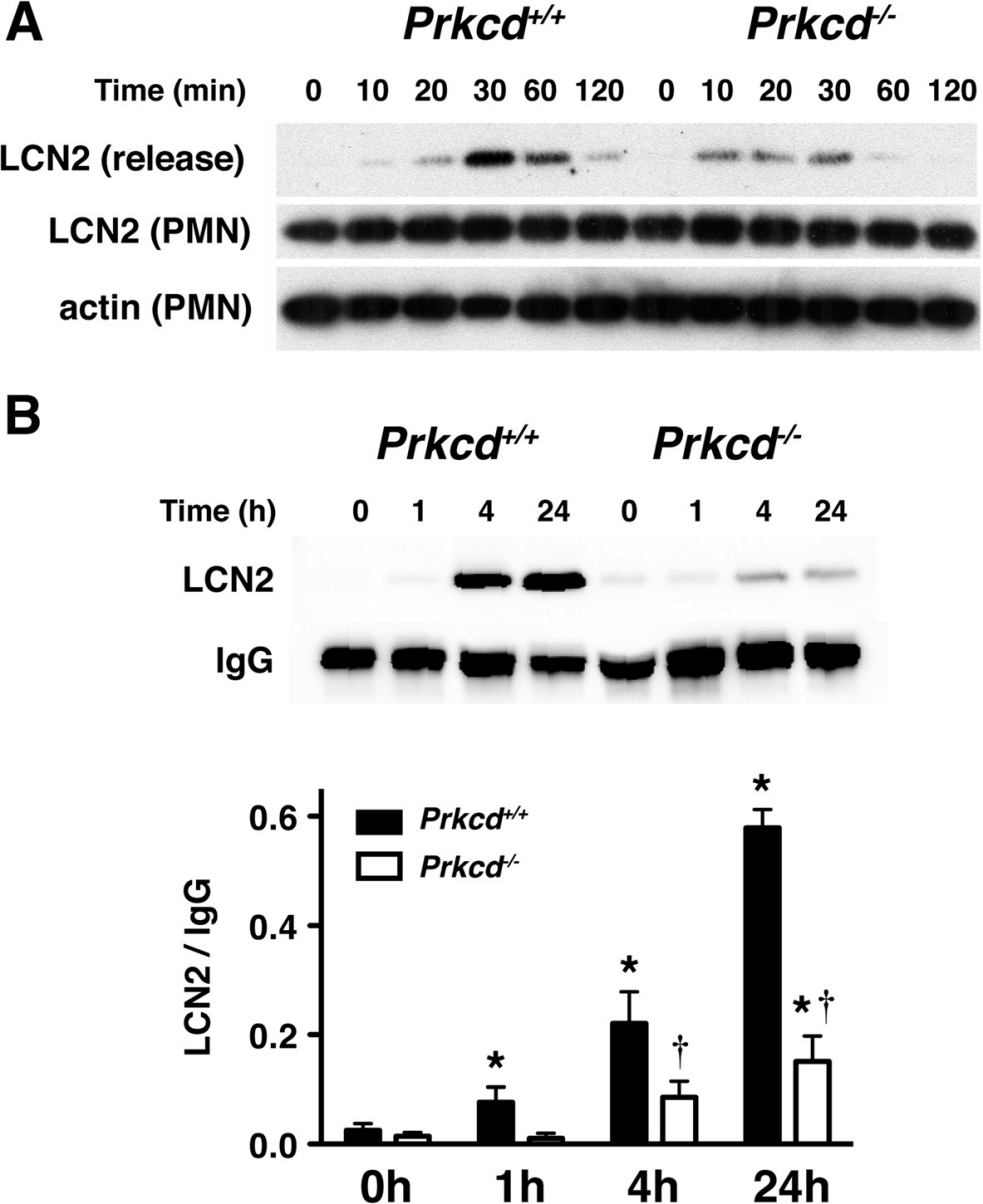
**The release of LCN2 is reduced in** ***Prkcd*** ^***−/−***^ **mice in response to fMLP and cerebral ischemia. (A)** Neutrophils from *Prkcd* ^*+/+*^ and *Prkcd* ^*−/−*^ were stimulated with 1 μM of fMLP at 37°C for up to 120 min. LCN2 released into the media (release) and remained in neutrophils (PMN) at different time points were detected by western blot analysis. Actin detected in neutrophils (PMN) was used as the loading control. **(B)** Mouse sera collected at different time points after global cerebral ischemia were subjected to western blot analysis with anti-LCN2 and anti-IgG antibodies. The serum of mice without ischemia was collected as a control (0). The top panel is a representative western blot. The LCN2 and IgG protein bands were quantified by densitometry in the bottom panel. Ischemia-induced LCN2 differed by genotype [F(1,22) = 56.6; *p* < 0.0001] and time [F(3,22) = 60.5; *p* < 0.0001] with an interaction between these factors [F(3,22) = 21.3; *p* < 0.0001]. * *p* < 0.05 compared with time 0 within genotype. † *p* < 0.05 compared with *Prkcd* ^*+/+*^ mice at the same time point (Bonferroni tests) (*n* = 3–5).

Previous studies demonstrated that the level of LCN2 in human plasma is elevated after ischemic stroke [15-17]. Since peripheral blood neutrophils are activated during the first few hours after stroke [2,44], the elevated LCN2 in plasma is likely derived from activated neutrophils. We previously found that several measures of neutrophil activation are impaired in neutrophils from *Prkcd*^*−/−*^ mice [4]. We therefore hypothesized that LCN2 secretion would be impaired as well. To induce LCN2 secretion *in vitro*, we treated neutrophils with fMLP and found that the fMLP-stimulated release of LCN2 was reduced in *Prkcd*^*−/−*^ neutrophils (Figure 6A). This result could not be explained by reduced abundance of LCN2, since LCN2 abundance was similar in wild type and *Prkcd*^*−/−*^ neutrophils (Figure 6A), suggesting that PKCδ is involved in regulating the secretion but not the expression of LCN2. We then examined the level of LCN2 in the sera of wild type and *Prkcd*^*−/−*^ mice after global cerebral ischemia (Figure 6B). Blood sera collected at different intervals after 10 min of bilateral common carotid artery occlusion (BCCAO) were subjected to western blot analysis for LCN2. The abundance of LCN2 immunoreactivity was low in the sera of mice not subjected to BCCAO. In wild type mice, the level of LCN2 increased one hour after BCCAO, and increased progressively further at 4 and 24 hours. LCN2 induction after BCCAO was greatly reduced in *Prkcd*^*−/−*^ mice, indicating a role of PKCδ in mediating LCN2 release after cerebral ischemia.

## Discussion

In this study, we employed a chemical-genetics approach to identify PKCδ substrates in neutrophils. One of the PKCδ substrates was LCN2, a member of the lipocalin family. PKCδ directly phosphorylates LCN2 at T115 *in vitro* and in neutrophils. The members of lipocalin family share little overall sequence homology, but all form a similar cup-shaped structure suited for carrying small hydrophobic molecules [36]. The crystal structure of LCN2 with nine β-strands and one α-helix forms a funnel-like binding pocket containing an iron-loaded siderophore [35] (Figure 3E). The PKCδ phosphorylation site (T115) is not located within the siderophore-binding pocket, but on the β5 strand with its side chain projecting outward. Thus, phosphorylation of T115 is unlikely to alter the binding with the siderophore, but instead could regulate the interaction of LCN2 with other molecules.

The anti-phospho-LCN2 (T115) immunoreactivity was significantly reduced in the neutrophil lysates of *Prkcd*^*−/−*^ mice (Figure 4). However, the presence of residual immunoreactivity in *Prkcd*^*−/−*^ mice suggests that T115 might also be phosphorylated by other kinases. There are three additional PKC isozymes (α, β, ζ) found expressed in neutrophils [4,42,43]. The preferred amino acid sequences flanking the phosphorylated residue for each PKC isozyme have been determined as isozyme specific phosphorylation motifs [45,46]. The four PKC isozymes (α, β, δ, ζ) in neutrophils all prefer phosphorylating peptides with a hydrophobic residue at position +1 of the phosphorylated site towards the carboxyl-terminal [45,46]. The amino acid following Thr-115 of LCN2 fits that criterion with Leu at this position (Figure 3B). PKCα and δ, but not PKCβ and ζ, prefer Arg at −5 position. Only PKCδ, but not other neutrophil PKC’s, prefers hydrophobic residues at +2, +3 and +4. The sequence flanking Thr-115 contains Arg at −5 and Met at +4, so Thr-115 may be preferentially phosphorylated by PKCδ. However, the sequence lacks a basic residue at the −3 position, and thus it is not fully conserved as a PKCδ specific phosphorylation motif. These linear phosphorylation motifs are short peptide sequences derived from *in vitro* kinase assays using an oriented peptide library [45,46]. Thus, predictions based on motifs are suggestive, but may not fully capture the phosphorylation events *in vivo*. In fact, recent studies demonstrate that PKC and PKA phosphorylate Ser/Thr residues of substrates within sequences that do not match known linear motifs [47]. The folding of different parts of the substrate can create “structurally formed” phosphorylation motifs. LCN2 exists as a monomer and homodimer as well as heterodimer with MMP-9 [36,48]. The tertiary and quaternary structures of LCN2 may contribute to the specificity of substrate phosphorylation by PKCδ. Future studies will be needed to decipher the mechanisms in detail.

We found that LCN2 is released from fMLP-stimulated neutrophils *in vitro* and into the serum after cerebral ischemia (Figure 6). Previous studies demonstrate that LCN2 is up-regulated in mouse models of spinal cord injury [14] and neurodegeneration [49]. Neutrophil infiltration, expression of pro-inflammatory chemokines and cytokines, and neuronal cell death after spinal cord injury [14] are reduced in LCN2 null mice. We recently found that brain injury, neurological deficits, and infiltration of immune cells were markedly diminished in LCN2 null mice when compared with wild type mice after stroke-reperfusion injury [50]. Recombinant LCN2 stimulates neutrophil migration *in vitro* and *in vivo* [51] and induces apoptosis by sequestering intracellular iron [21,34]. Moreover, stroke patients with higher LCN2 levels in blood plasma show higher cardiovascular mortality [17]. These findings suggest that LCN2 is a chemoattractant for neutrophils and a pro-inflammatory signal induced by nervous system injury. Our previous study shows that neutrophil infiltration and brain injury are reduced in *Prkcd*^*−/−*^ mice after ischemic stroke [4]. Given our current results showing that LCN2 release is reduced in *Prkcd*^*−/−*^ mice, the mild stroke phenotype in *Prkcd*^*−/−*^ mice may result in part from reduced release of LCN2. Taken together, our results suggest that reduction of PKCδ activation and LCN2 release might prove useful in reducing post-ischemic inflammation and brain injury after stroke.

## Conclusions

Neutrophil PKCδ contributes to stroke-reperfusion injury, but the underlying mechanisms remain to be determined. In this report, we identified lipocalin-2 as a PKCδ substrate in neutrophils using a chemical-genetics approach. PKCδ phosphorylates lipocalin-2 at T115 and mediates the secretion of lipocalin-2 in neutrophils. This study provides a potential mechanism for the role of PKCδ in stroke-reperfusion injury.
